# Multigene Phylogenetics and Morphology Reveal Five Novel *Lasiodiplodia* Species Associated with Blueberries

**DOI:** 10.3390/life11070657

**Published:** 2021-07-05

**Authors:** Yu Wang, Ying Zhang, Vishwakalyan Bhoyroo, Sillma Rampadarath, Rajesh Jeewon

**Affiliations:** 1 School of Ecology and Nature Conservation, Beijing Forestry University, P.O. Box 61, Beijing 100083, China; yinghku@gmail.com; 2 Forest Pest Control and Quarantine Station of Hebei Province, Shijiazhuang 050081, China; 3 Faculty of Agriculture, University of Mauritius, Reduit 80837, Mauritius; v.bhoyroo@uom.ac.mu; 4 Charles Regnaud Street, Curepipe 74319, Mauritius; sillma.rampadarath@gmail.com; 5 Department of Health Sciences, Faculty of Medicine and Health Sciences, University of Mauritius, Reduit 80837, Mauritius

**Keywords:** Botryosphaeriaceae, fruit tree, stem disease, taxonomy, *Vaccinium* spp.

## Abstract

Botryosphaeriaceous fungi cause stem blight, canker and dieback in woody plants. During a survey on the fungal pathogens associated with blueberries in China, 135 blighted, cankered or dead blueberry branches were collected from Fujian and Shandong Provinces. Based on the morphological characterization and phylogenetic analyses of a concatenated ITS rDNA, *tef1-α*, *TUB*, and *RPB2* loci, five new species of *Lasiodiplodia*, viz., *L*. *clavispora*, *L*. *fujianensis*, *L*. *henanica*, *L*. *nanpingensis* and *L*. *paraphysoides* were recognized. Detailed descriptions and illustrations, as well as multigene phylogenies, are provided in this paper. The diversity of plant pathogens on agriculturally and economically important plants is higher than anticipated.

## 1. Introduction

Blueberries (*Vaccinium* spp.) are perennial shrub fruit trees. The fruits are well known and are widely consumed for their protective properties against heart diseases and cancer, they can help to maintain bone strength and mental health and can regulate blood pressure [1]. Blueberries are widely distributed in temperate regions, such as North America, Europe, Canada and Northern China [2,3,4,5,6,7,8,9,10,11,12,13]. Due to their health benefits and economic value, blueberries have been commercially cultivated worldwide, particularly in the USA, Canada and European countries [14,15]. Blueberry cultivation started in 1981 in China, and productivity has reached 43,244 tons per year [16].

Botryosphaeriaceous fungi are a group of economically important plant pathogens [17,18,19,20]. They cause stem blight, canker or dieback in a wide range of hosts, including blueberries [8,9,11,15,21,22,23,24,25]. In the USA, blueberry stem blight, caused by *Botryosphaeria* *ribis*, has been a major disease in commercial plantations in North Carolina [26,27]. Pathogenicity studies conducted show that stem dieback is caused by *B*. *dothidea* and canker by *Lasiodiplodia corticis* in blueberries in North Carolina. *Neofusicoccum parvum* was identified as the causal agent for blueberry stem blight and dieback in California and Mexico [2,4]. In Florida, the blueberry stem blight and dieback caused by *Neofusicoccum ribis* and *Lasiodiplodia theobromae* led to huge economic losses and were one of the most severe diseases in the local blueberry planting industry [8,9,28]. The incidence of blueberry stem blight and canker caused by *Neofusicoccum parvum* has been a limiting factor for blueberry production in Chile [29]. The incidence of blueberry blight and crown rot caused by *N*. *ribis* and *L*. *theobromae* was so severe in New Zealand that it resulted in an annual loss of about USD 500,000 due to yield losses and replanting [6,8,9,28]. *Neofusicoccum parvum* and *N*. *austral* caused blueberry stem dieback and canker in Spain [3,30]. Many more botryosphaeriaceous species have been reported to cause blueberry stem dieback or canker worldwide, such as *Botryosphaeria corticis*, *Lasiodiplodia mediterranea*, *L*. *pseudotheobromae*, *Macrophomina phaseolina*, *Neofusicoccum arbuti*, *N*. *austral*, *N*. *kwambonambiense*, *N*. *macroclavatum*, *N*. *occulatum* and *N*. *ribis* [7,8,9,15,29,31,32,33,34].

Blueberry cultivation started in 1981 in China and, subsequently, the blueberry stem diseases caused by botryosphaeriaceous fungi received more and more attention. For instance, some studies first reported that blueberry bud and stem blight or dieback were caused by *Neofusicoccum vitifusiforme* in Yunnan Province in China [5,12]. In addition, *N. parvum* caused blueberry stem blight in highbush blueberries (*Vaccinium corymbosum*) in Yunnan Province [12]. In Shandong Province, it was reported that blueberry stem blight and dieback were caused by *Lasiodiplodia pseudotheobromae* (current name *L*. *chinensis*) [22,35]. It was noticed that botryosphaeriaceous fungi cause blueberry stem blight or dieback in eight provinces in China, and three species were recognized: *Botryosphaeria dothidea*, *Lasiodiplodia theobromae* and *Neofusicoccum parvum* [11]. Furthermore, it was also noticed that *L*. *theobromae* and *N*. *parvum* are more virulent than *Botryosphaeria dothidea* [11]. A new fungus has been described, viz. *L*. *vaccini*, which causes blueberry stem blight in the greenhouses of blueberry plantations in rural areas of Beijing [13]. The pathogenicity of *Botryosphaeriaceae* was discussed by Manawasinghe et al. [36].

During a survey on the fungal pathogens associated with blueberries in China, several species of *Lasiodiplodia* have been identified, and five of them are described as new to science. A concatenated DNA dataset from ITS rDNA and *tef1-α*, *TUB*, and *RPB2* loci have been analyzed, and the phylogenetic relationships of these novel species have been established.

## 2. Methods and Materials

### 2.1. Sample Collections and Fungal Isolation

One hundred and thirty-five blighted, cankered or dead blueberry branches were collected from Fujian (69 samples) and Shandong (66 samples) Provinces in China from April to November 2018. Diseased or dead twigs of blueberries (ca. 30 cm) were cut for sampling, from which the fungal strains were isolated. Wood segments (0.5 × 0.5 × 0.2 cm) were cut from the diseased lesion boundary or dead tissues and were subsequently surface sterilized and incubated in malt extract agar (MEA) at 28 °C for fungal strain isolation [13,37,38,39]. The isolates were kept at ambient temperatures (about 26–28 °C) and grown in the dark.

### 2.2. Morphological Characterization

Fungal colonies were initially identified based on morphological characteristics. Fungal isolates were transferred to synthetic nutrient-poor agar (SNA) with sterilized pine needles for 3 weeks in order to induce sporulation. The pycnidia produced on the pine needles were morphologically described following the work by Dou et al. [35,40]. Microscopic observations were made from material mounted in water. Measurements of paraphyses, conidiogenous cells and conidia were made in water. For each new species, the measurements of 20 paraphyses, 20 conidiogenous cells and 50 conidia were taken under a Nikon Eclipse E600 microscope. Fungal isolates and herbarium specimens were deposited at the China General Microbiological Culture Collection Center (CGMCC) and the Mycological Herbarium of the Institute of Microbiology, Chinese Academy of Sciences (HMAS). The new species were established based on the guidelines outlined by Jeewon and Hyde [41].

### 2.3. DNA Extraction, PCR Amplification

DNA was extracted with the CTAB plant genome DNA fast extraction kit (Aidlab Biotechnologies Co, Ltd., Beijing, China) from the mycelium grown on MEA. PCR amplifications were performed using the Easy Taq PCR Super Mix kit (Beijing Transgen Biotech Co., Ltd., Beijing, China). The internal transcribed spacers of rDNA (ITS) were amplified and sequenced with the primers ITS-1 and ITS-4 [42]. The translation elongation factor-1α (*tef1-α*) was amplified and sequenced with primers EF1-688F and EF1-1251R [43]. The *TUB* gene was amplified and sequenced with primers Bt2a and Bt2b [44]. The *RPB2* were amplified and sequenced using primers *RPB2*-LasF and *RPB2*-LasR [45]. PCR amplification and sequencing followed the protocol outlined by Zhang et al. [46]. PCR amplifications were performed using the Easy Taq PCR Super Mix kit (Beijing Transgen Biotech Co., Ltd., Beijing, China). For the ITS regions, the following PCR profile was used: 95 °C for 3 min, followed by 34 cycles of denaturation at 95 °C for 1 min, annealing at 52°C for 30 s and elongation at 72 °C for 1 min, with a final extension step of 72 °C for 10 min. The PCR profiles for the *tef1-α*, *TUB* and RPB2 genes were same, except that 35 cycles of denaturation were used and the annealing temperature was 55 °C.

### 2.4. Sequence Alignment and Phylogenetic Analysis

The concatenated loci of ITS, *tef1-α*, *TUB* and *RPB2* were used to infer the phylogenetic relationships of taxa within *Lasiodiplodia*. Alignments were conducted in MEGA v. 6, and phylogenetic analyses performed in PAUP v. 4.0b10 and MrBayes v. 3.1.2 [47,48,49]. Prior to phylogenetic analysis, ambiguous sequences at the start and end were deleted and gaps manually adjusted in order to optimize the alignments. Maximum Parsimony (MP) was used to conduct heuristic searches, as implemented in PAUP with the default options method [50]. Analyses were conducted under different parameters of maximum parsimony criteria [50]. Clade stability was assessed in a bootstrap analysis with 1000 replicates, random sequence additions with maxtrees set to 1000 and other default parameters, as implemented in PAUP. For the MrBayes analysis, the best-fit model of nucleotide evolution (GTR+I+G) was selected by the Akaike information criterion [51] in MrModeltest v. 2.3. The metropolis-coupled Markov Chain Monte Carlo (MCMCMC) approach was used to calculate posterior probabilities [47]. Bayesian inference (BI) analysis with MrBayes revealed that the Markov chain Monte Carlo (MCMC) steady state was reached after fewer than 19,820,000 generations (the average standard deviation of split frequencies was constantly below 0.01). A conservative burn-in of 198,200 trees was chosen, and a full analysis of 20,000,000 generations was carried out with sampling every 100 generations. Trees were viewed in TREEVIEW [52]. The nucleotide sequences generated in this paper were deposited in GenBank (Table 1). Trees and alignments were deposited in TreeBase (http://purl.org/phylo/treebase/phylows/study/TB2:S24322?x-access-code=1443788eea51ad240fcd94b3927ffb1a&format=html, accessed on 15 June 2021).

## 3. Results

**Figure 1:**
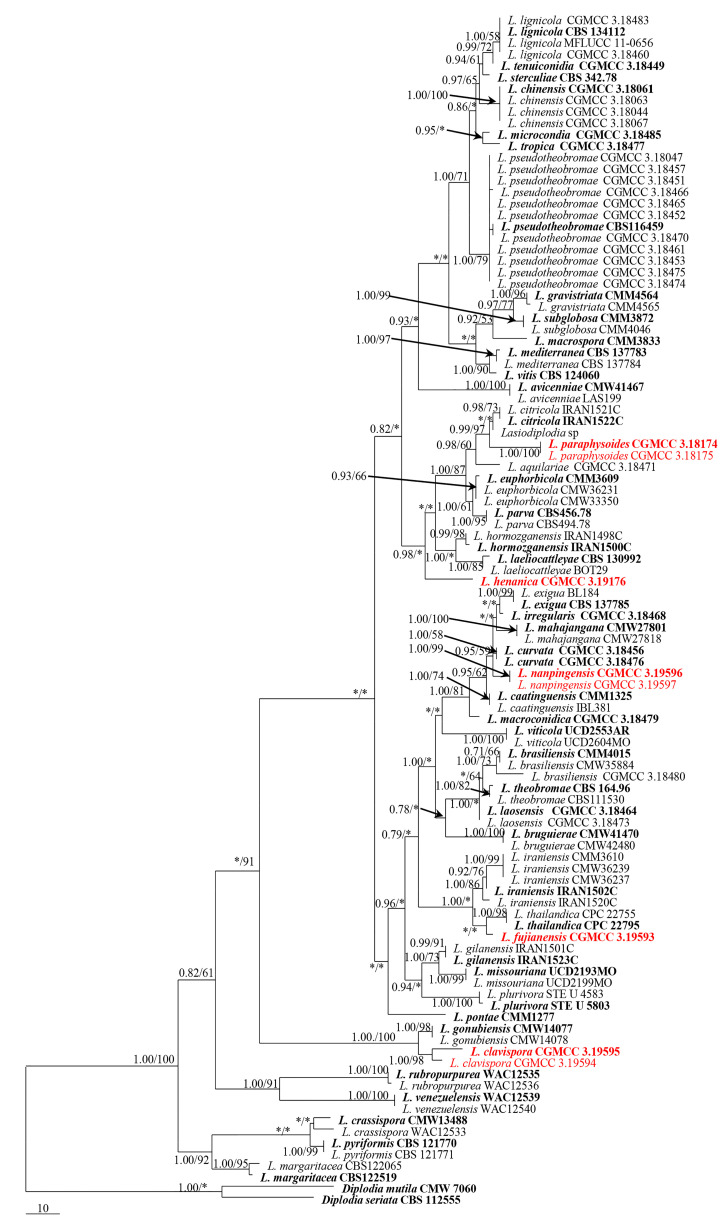
Maximum parsimony tree obtained from combined sequence ITS nrDNA, *tef1*-α, *TUB* and *RPB2* dataset of *Lasiodiplodia* species. Designated out-group taxon is *Diplodia mutila* (CMW 7060) and *D. seriata* (CBS 112555). Bayesian posterior probabilities (PP) support the above 0.7 and maximum parsimony (MP) support values above 50%, are shown on nodes (PP/MP). * represents either PP or MP support values which are below 0.7 (PP) and 50% (MP) respectively. Ex-type strains are printed in bold face and new isolates in red bold face.

For *Lasiodiplodia*, the concatenated ITS, *tef1-α*, *TUB*, and *RPB2* DNA sequence dataset comprises 1974 bp with 335 parsimony-informative characters. A MP tree (TL = 890 steps, CI = 0.609, RI = 0.870, RC = 0.530, HI = 0.391) generated based on a heuristic search with the random addition of taxa (1000 replicates) is shown in Figure 1.

**Taxonomy**

**Figure 2:**
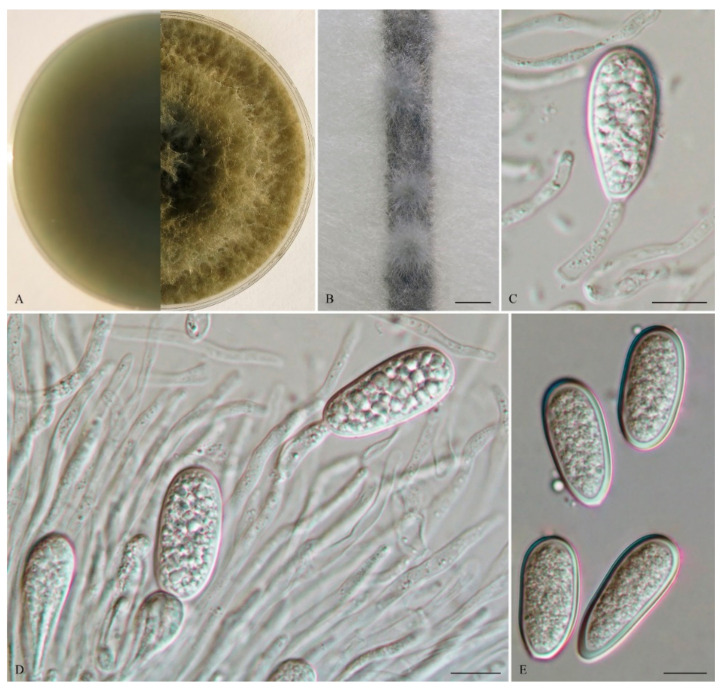
*Lasiodiplodia clavispora* (From holotype HMAS 255607). (**A**). Culture grown on MEA. (**B**). Conidiomata developing on pine needles in culture. (**C**,**D**). Conidia developing on conidiogenous cells between paraphyses. (**E**). Hyaline, aseptate conidia. Scale bars: B = 1 mm; C–E = 10 µm.

*Lasiodiplodia clavispora* Y. Zhang ter., Y. Wang, sp. nov. (Figure 2).

MycoBank: MB 830994.

The etymology of the name reflects the clavate conidia.

The sexual stage was not observed. *Conidiomata* were stromatic, produced on both sterilized pine needles and SNA within 10 days, it was semi-immersed, uniloculate and rarely multiloculate, black, covered by greyish brown mycelium, and up to 570 μm diam when there was uniloculate. *Paraphyses* were filiform, arising from the conidiogenous layer, extending above the level of developing conidia, up to 100 μm long and 3 μm wide, cylindrical, thin walled, aseptate, hyaline, tip rounded, and unbranched. *Conidiophores* were reduced to conidiogenous cells. *Conidiogenous cells* were holoblastic, hyaline, discrete, smooth, and thin-walled, (9.5–) 11–18 (–19) × 2.5–5 μm (mean of 50 conidiogenous cells = 14.3 × 3.8 μm, L/W ratio = 4). *Conidia* were hyaline, with a wall of 1–2 μm thick, clavate, narrowly ellipsoid to narrowly ovoid with a round apex and had a slightly tapered base, (28–) 29–36 (–38) × 12–15 μm (mean of 50 conidia = 31.7 × 13.8 μm, L/W ratio = 2.3, range from 2.0 to 3.0), no pigmented conidia observed after 15 days. *Spermatia* were not observed.

*Culture characteristics*: Colonies on MEA were initially white with moderately dense aerial mycelia reaching the lid of the plate and became olive grey on the surface after 5 d, with the reverse side of the colonies being pale grey to grey. Colonies reached 18 mm on MEA after 24 h in the dark at 28 °C, and were more than 55 mm after 48 h.

*Materials examined:* CHINA, Fujian province, Nanping, Jianyang district, from blighted stems of *Vaccinium uliginosum* Linn., 1 April 2018, L. Zhao (Holotype: HMAS 255607, ex-type isolate: CGMCC 3.19594; Paratype: HMAS 255612, isolate: CGMCC 3.19595).

*Notes:* Phylogenetically, *L*. *clavispora* is closely related to *L*. *gonubiensis* (PP/MP = 1.00/100, Figure 1). *Lasiodiplodia clavispora* (CGMCC 3.19594) differs from its closest phylogenetic neighbor *L*. *gonubiensis* (CMW14077) (Figure 1) by 14 bp in *tef1-α* (0.72 %) (Table 2). In addition, a conidial size of *L*. *clavispora* also differs from *L*. *gonubiensis* (12–15 vs. (14–) 16–18.5 (–21) μm) [37].

**Figure 3:**
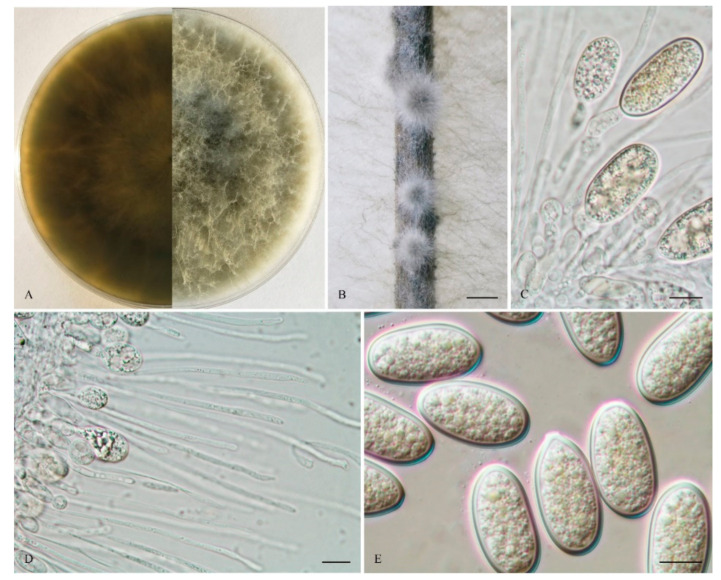
*Lasiodiplodia fujianensis* (From holotype HMAS 255606). (**A**). Culture grown on MEA. (**B**). Conidiomata developing on pine needles in culture. (**C**). Conidia developing on conidiogenous cells between paraphyses. (**D**). Aseptate and unbranched paraphyses. (**E**). Hyaline and aseptate conidia. Scale bars: B = 1 mm; C–G = 10 µm.

*Lasiodiplodia fujianensis* Y. Zhang ter., Y. Wang, sp. nov (Figure 3).

MycoBank: MB 830996.

The etymology is in reference to the location, Fujian province, where the species was first reported.

The sexual stage was not observed. Conidiomata were stromatic, produced on both sterilized pine needles and SNA within 10 days, semi-immersed, uniloculate, black, covered by greyish brown mycelium, and were up to 1.3 mm in diameter. *Paraphyses* were filiform, arising from the conidiogenous layer that extended above the level of developing conidia and were up to 95 μm long and 3 μm wide, aseptate, hyaline, tip rounded, and unbranched. *Conidiophores* reduced to conidiogenous cells. *Conidiogenous* cells were holoblastic, hyaline, discrete, smooth, and thin walled, (11–) 12–18.5 (–20) × (3–) 4–8 (–8.5) μm (mean of 50 conidiogenous cells = 14.9 × 5.4 μm, L/W ratio = 2.9). *Conidia* were hyaline, with a 1–2 μm thick wall, ellipsoid with a round apex and round base, and occasionally truncated at the base, (22–) 23–29 (–30) × (12–) 13–15 (–16) μm (mean of 50 conidia = 26.2 × 14.5 μm, L/W ratio = 1.8, range from 1.5 to 2.2), with pigmented conidia observed after 15 days. *Spermatia* were not observed.

*Culture characteristics:* Colonies on MEA were initially white with moderately dense aerial mycelia reaching the lid of the plate and became ash-grey on the surface after 5 d, with the reverse side of the colonies being pale grey to grey. Colonies reached 45 mm on MEA after 24 h in the dark at 28 °C, and more than 90 mm after 48 h.

*Materials examined:* China, Fujian Province, Nanping, Jianyang district, from blighted stems of *Vaccinium uliginosum*, 1 April 2018, L. Zhao (Holotype: HMAS 255606, ex-type isolate: CGMCC 3.19593).

*Notes:* Phylogenetically, *L*. *fujianensis* is basal to *L*. *thailandica* and *L*. *iraniensis* (Figure 1). *Lasiodiplodia fujianensis* (CGMCC 3.19593), however, differs from *L*. *thailandica* (CPC 22755) (Figure 1) by 16 bp in *tef1-α* (1.09 %, gaps included) (Table 2). Morphologically, *L*. *fujianensis* also differs from *L*. *thailandica* in the size of the conidiomata and conidiogenous cells (310–330 × 300–370µm and 8–9 × 2–4 µm, respectively [53]. In addition, the aseptate paraphyses of *L*. *fujianensis* also make it morphologically different from *L*. *thailandica* (1–3-septate).

**Figure 4:**
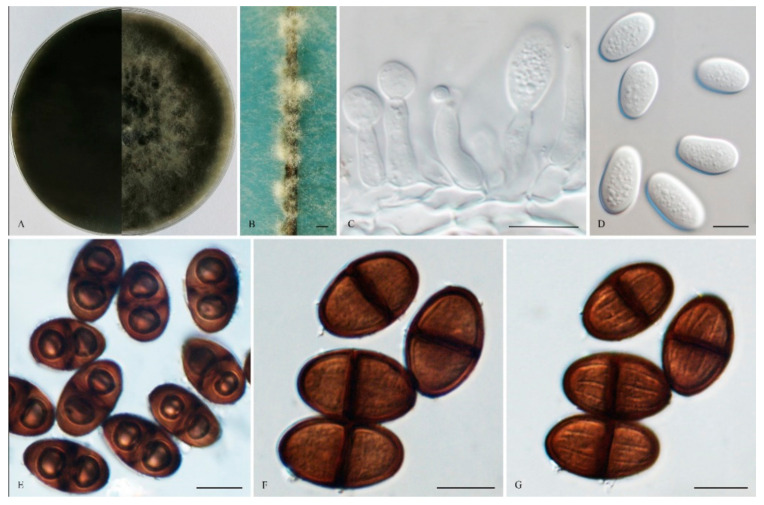
*Lasiodiplodia henanica* (from holotype HMAS 247961). (**A**). Culture grown on MEA. (**B**). Conidiomata developing on pine needles in culture. (**C**). Conidia developing on conidiogenous cells. (**D**). Hyaline and immature conidia with granular content. (**E**). Conidia with two vacuoles. (**F**,**G**) Pigmented, 1-septate conidia in two different focal planes to show the longitudinal striations. Scale bars: B = 1 mm; C–G = 10 µm.

*Lasiodiplodiahenanica* Z. P. Dou, Y. Wang, Y. Zhang ter. sp. nov. (Figure 4).

Mycobank: MB 817650.

The etymology is in reference to the location, Henan province, where the species were reported.

The sexual stage was not observed. *Conidiomata* were stromatic, produced on both sterilized pine needles on SNA within 14 days, and were semi-immersed or superficial, mostly solitary, globose, smooth, mostly non-papillate, iron grey to black, covered by brown mycelium, and up to 520 μm in diameter. *Paraphyses* were filiform and arose from the conidiogenous layer, extending above the level of developing conidia, and were up to 105 μm long and 4 μm wide, cylindrical, thin-walled, initially aseptate, which became up to 1–3-septate when mature, hyaline, apex rounded, occasionally basal cells swollen, and unbranched. *Conidiophores* were reduced to *Conidiogenous* cells. *Conidiogenous* cells were holoblastic, hyaline, discrete, smooth, thin-walled, and were cylindrical to ampulliform, (8–) 9–16 × 3 –5 (–7) μm (mean of 50 conidiogenous cells = 12.1 × 4.0 μm, L/W ratio = 2.95). *Conidia* were initially hyaline, with a 1 μm thick wall, ovoid to cylindrical, turning brown with a median septum and longitudinal striations when mature, and sometimes with two vacuoles, (14–) 19–26 (–27) × 10–13 (–15) μm (mean of 100 conidia = 22.1 × 12.0 μm, L/W ratio = 1.86, by range from 1.17 to 2.6). *Spermatia* were not observed.

*Culture characteristics:* Colonies on MEA were initially white with moderately dense aerial mycelia reaching the lid of the plate and became dark grey to black on the surface after 7 d, with the reverse side of the being colonies dark black. Colonies reached 26 mm on MEA after 24 h in the dark at 28 °C, and more than 65 mm after 48 h.

*Specimens examined:* China, Shandong province, Qingdao, Huangdao district, were from blighted stems of *Vaccinium uliginosum*, 17 November 2018, Y. Zhang and L. Zhao (Holotype: HMAS 247961, ex-type isolate: CGMCC 3. 19176). Henan province, Puyang city, Qingfeng, a farmer orchard was from cankered stems of *Morus alba* Linn. var. alba, 11 November 2014, Z. P. Dou & W. He (Paratype: HMAS 255410, isolate: CGMCC 3.17969).

Notes: Phylogenetically, *L. henanica* is basal to the clade and comprised of *L. citricola*, *L. paraphysoides*, *L. aquilariae*, *L. euphorbicola*, *L. parva*, *L. hormozganensis* and *L. laeliocattleyae*. Morphologically, *L. henanica* differs from *L. hormozganensis* in having smaller-sized conidiomata (up to 520 μm vs. up to 950 μm) [54]. In addition, the presence of vacuoles in the conidia of *L. henanica* also makes it different from *L. hormozganensis* and *L. laeliocattleyae* [54,55]. The broader 1–3-septate paraphyses of *L. henanica* are also distinguishable from *L. laeliocattleyae* (up to 3 μm, aseptate) [55].

**Figure 5:**
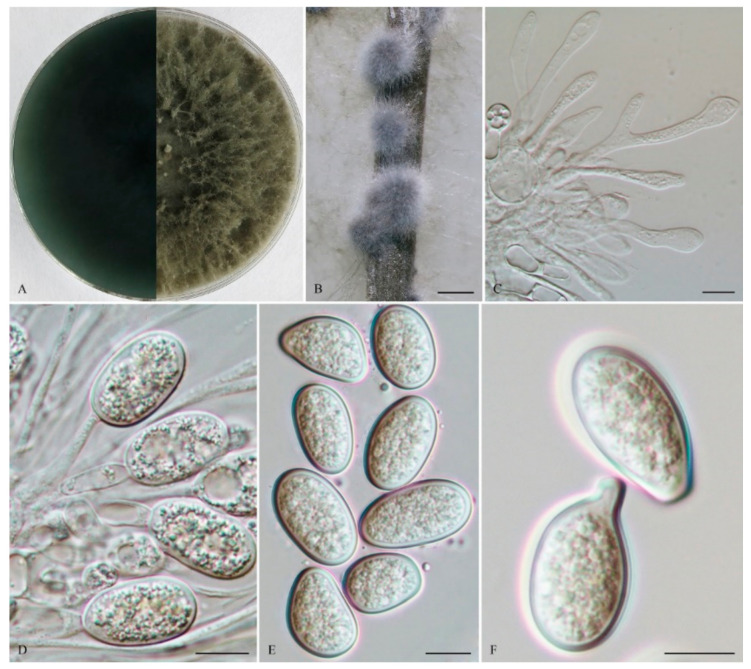
*Lasiodiplodia nanpingensis* (from holotype HMAS 255608). (**A**). Culture grown on MEA. (**B**). Conidiomata developing on pine needles in culture. (**C**). Developing, aseptate and branched paraphyses. (**D**). Conidia developing on conidiogenous cells between paraphyses. (**E**). Hyaline, aseptate conidia. (**F**). Germinating conidia. Scale bars: B = 1 mm; C–F = 10 µm.

*Lasiodiplodia nanpingensis* Y. Zhang ter., Y. Wang, sp. nov. (Figure 5).

MycoBank: MB 830997.

The etymology of the name reflects Nanping, where this species was first reported.

The sexual stage was not observed. *Conidiomata* were stromatic, it was produced on both sterilized pine needles and SNA within 7 days, and was solitary, scattered or in small groups (up to 5), semi-immersed or superficial, uniloculate, black, covered by greyish brown mycelium, and up to 640 μm diam. *Paraphyses* were filiform, arising from the conidiogenous layer, extending above the level of developing conidia, up to 102 μm long and 3.5 μm wide, and was aseptate, hyaline, tip rounded, and branched. *Conidiophores* was reduced to conidiogenous cells. *Conidiogenous* cells were holoblastic, hyaline, discrete, smooth, and thin walled, 9–16 (–19) × 3–6 (–7) μm (mean of 50 conidiogenous cells = 13.0 × 4.6 μm, L/W ratio = 2.97). *Conidia* were hyaline, with a 1 μm thick wall, ellipsoid with round apexes and was rarely irregular, (20–) 21–26 (–28) × 13–16 (–17) μm (mean of 50 conidia = 23.9 × 14.8 μm, L/W ratio = 1.6, range from 1.4 to 1.9). *Spermatia* were not observed.

*Culture characteristics:* Colonies on MEA were initially white with moderately dense aerial mycelia reaching the lid of the plate and becoming ash-grey on the surface after 5 d, with the reverse side of the colonies being pale grey to grey. Colonies reached 17 mm on MEA after 24 h in the dark at 28 °C, and more than 60 mm after 48 h.

*Materials examined:* China, Fujian province, Nanping, Jianyang district, from blighted stems of *Vaccinium uliginosum*, 1 April 2018, L. Zhao (Holotype: HMAS 255608, ex-type isolate: CGMCC 3.19596; Paratype: HMAS 255609, isolate: CGMCC 3.19597).

*Notes:* Phylogenetically, the clade comprising *L*. *curvata*, *L*. *exigua*, *L*. *mahajangana*, *L*. *nanpingensis* and *L*. *irregularis* received moderate bootstrap support (PP/MP = 0.95/59) (Figure 1). It can also be noted that our two strains of *L*. *nanpingensis* constituted a strongly supported independent subclade. Morphologically, the deeply curved conidia of *L*. *curvata* distinguished it from *L*. *nanpingensis* [56]. The larger-sized conidiomata of *L*. *nanpingensis* also differed from *L*. *irregularis* (up to 640 μm vs. up to 400 μm). In addition, the branched and aseptate paraphyses of *L*. *nanpingensis* made the latter distinct from the unbranched and 1-septate paraphyses of *L*. *irregularis* [56], as well as from *L*. *mahajangana* [57]. The larger-sized conidiomata and conidia of *L*. *nanpingensis* also differed from *L*. *mahajangana* [57]. *Lasiodiplodia nanpingensis* became longer and had slender paraphyses, which were different from those of *L*. *exigua* (up to 102 × 3.5 μm vs. up to 66 × 5 μm) [58].

**Figure 6:**
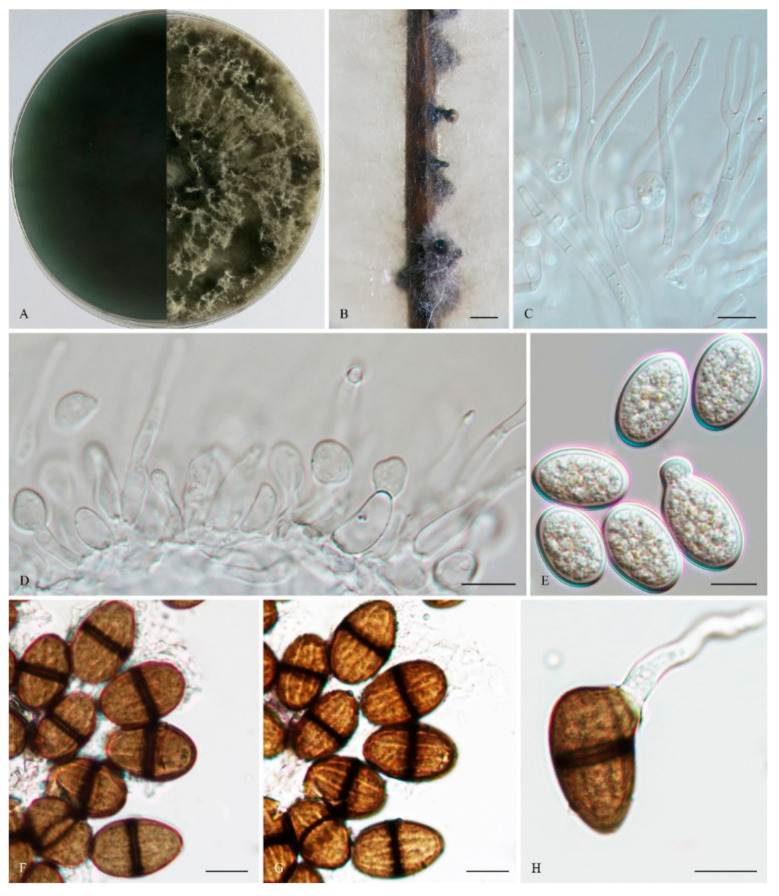
*Lasiodiplodia paraphysoides* (From holotype HMAS 247959). (**A**). Culture grown on MEA. (**B**). Conidiomata developing on pine needles in culture. (**C**). Septate or aseptate, unbranched or branched paraphyses. (**D**). Conidia developing on conidiogenous cells between paraphyses. (**E**). Hyaline, immature and germinating conidia. (**F**,**G**). Pigmented, 1-septate conidia in two different focal planes to show the longitudinal striations. (**H**). Germinating pigmented conidia. Scale bars: B = 1 mm; C–H = 10 µm.

*Lasiodiplodia paraphysoides* Z. P. Dou, Y. Wang, Y. Zhang ter sp. nov. (Figure 6).

Mycobank: MB 817655.

The etymology is in reference to the long and multiseptate paraphyses.

The sexual stage was not observed. *Conidiomata* were stromati, produced on both sterilized pine needles on SNA within 14 days, and were solitary, globose, semi-immersed or superficial, uniloculate, dark brown to black, covered with brown mycelium, up to 1.8 mm diam, and often had a long papilla, which was up to 383 μm long and 113 μm wide. *Paraphyses* were filiform, arising from the conidiogenous layer, extending above the level of developing conidia, up to 125 μm long and 7 μm wide, and were cylindrical, thin-walled, hyaline, tip rounded, initially aseptate, becoming up to 1–2-septate when mature, branched, occasionally basal, and were middle or apical swollen cells. *Conidiophores* were reduced to conidiogenous cells. *Conidiogenous* cells were holoblastic, hyaline, discrete, smooth, thin-walled, and were cylindrical to ampulliform, (8–) 10–16 (–18) × 3–7 μm (mean of 50 conidiogenous cells = 13.0 × 4.7 μm, L/W ratio =2.92). *Conidia* were initially hyaline, aseptate, with a 1 μm thick wall, and ellipsoid to ovoid with a round apex and round base, straight to obvious curved, turning brown with a median septum and longitudinal striations when mature, 1-septate, verruculose, (20–) 21–25 (–30) × (10–) 12–15 (–17) μm (mean of 50 conidia = 23.0 × 13.7 μm, L/W ratio = 1.69, range from 1.38 to 2.31), conidia sometimes germinating before septum formed or after pigmented. *Spermatia* were not observed.

*Culture characteristics:* Colonies on MEA were initially white with moderately dense aerial mycelia reaching the lid of the plate, and became dark grey on the surface after 7 d, with the reverse sides of the colonies dark grey to dark bluish grey. Colonies reached 20.5 mm on MEA after 24 h in the dark at 28 °C.

*Specimens examined:* China, Shandong province, Qingdao, Huangdao district, from blighted stems of *Vaccinium uliginosum*, 17 November 2018, Y. Zhang and L. Zhao (Holotype: HMAS 247959, ex-type isolate: CGMCC 3. 19174; Paratype: HMAS 247960, isolate: CGMCC 3. 19175).

*Notes:* Phylogenetically, *L*. *paraphysoides* was closely related to *L*. *citricola* and an unidentified taxon, *Lasiodiplodia* sp. *Lasiodiplodia paraphysoides* (CGMCC 3. 19174) and differred from its closest phylogenetic neighbor *L*. *citricola* (IRAN1522C) (Figure 1) by unique fixed alleles in two loci based on alignments of the separate loci deposited in TreeBASE (S25538), by 4 bp in *tef1-α* (0.72 %, gaps included) (Table 2). Morphologically, the long papilla of the conidiomata of *L*. *paraphysoides* delineated itself from the non-papillate conidiomata of *L*. *citricola* [54]. Furthermore, the conidiogenous cells of *L*. *citricola* had 1–2 annellations, which also differed from the holoblastic conidiogenous cells of *L*. *paraphysoides* [54].

## 4. Discussion

In this study, we recovered five new species of *Lasiodiplodia* associated with stem blight and/or canker of blueberries, namely, *L*. *clavispora*, *L*. *fujianensis*, *L*. *henanica*, *L*. *nanpingensis* and *L*. *paraphysoides*, and they were characterized in terms of their morphology and their phylogenetic relationships to other species of *Lasiodiplodia*. Phylogenetically, each of these five newly described species formed a well-supported subclade close to other species (Figure 1). Species of *Lasiodiplodia* were mostly differentiated based on the morphology of the conidia (especially dimensions) and paraphyses [17,35]. In this study, we attempted to use other morphological characters, such as the dimensions and papillate nature of conidiomata, as well as annelations of conidiogenous cells, but to what extent these are phylogenetically significant warrants further investigation.

Geographically, *Lasiodiplodia* tends to be distributed in tropical or subtropical areas or in warm temperate areas associated with various stem diseases of woody substrates [8,9,11,22,33,35]. For instance, *L*. *mediterranea* and *L*. *pseudotheobromae* have been reported as canker-causing agents of grapevine and other woody hosts in Italy, Algeria and Tunisia [58]. The stem blight and crown rot of blueberry caused by *L*. *theobromae* have been reported in Florida in the USA, as well as in Zhejiang Province and Shanghai in China [8,9,10,11]. The cane dieback of blueberry caused by *L*. *mediterranea* has been reported in Washington in the USA [33]. In China, blueberry stem blight and dieback caused by *L*. *chinensis* have been reported in Shandong Province [40,56]. The stem blight of blueberry caused by *L*. *vaccinii* was reported in a greenhouse plantation in Beijing, where it was warm with high humidity [13]. All the five species of *Lasiodiplodia* newly described in this study were from Fujian and Shandong Province, which belong to subtropical or warm temperate areas in China. The distribution of *Lasiodiplodia* spp seems largely influenced by environmental conditions, such as temperature, humidity, elevation, as well as the prevalence of alternative hosts instead of their host associations [28,59].

We also compared our species with newly described species recently published by de Silva et al. [60]. From a phylogenetic perspective, our new species are quite different, except for one, *L. fujianensisis*. The latter is basal to *L. thailandica* and *L. iraniensis*, which are known species. de Silva et al. [60] also reported that their new species, *L. endophytica* from *Magnolia* plant, are phylogenetically closely related to *L. thailandica* and *L. iraniensis* albeit in a distinct independent lineage with weak support. To avoid any ambiguous taxonomic interpretation in connection with the identification of *L. fujianensisis*, we compared DNA base pair differences with *L. endophytica*. DNA sequences from the TEF protein coding region for *L. endophytica* is quite short (271 bp) and we still found two major differences, which supports that our species is different. With respect to the DNA sequences from the Beta tubulin gene region, *L. fujianensisis* was 100% similar to *L. endophytica*. Could this be pointing to the fact that these two taxa are conspecific? This might be true, but we compared existing DNA sequences of the Beta tubulin from other published species, such as *L. pseudotheobromae*, *L. jatrophicola*, *L. vitis* and *L. iraniensis* and found that they are identical to *L. fujianensisis* and *L. endophytica*. The taxonomy of *Lasioplodia* has been rather controversial [35]. While some are proponents of a taxonomy based on morphological characteristics, others argue that more protein genes should be included in the taxonomy, especially at the species level. However, the protein genes might not be useful, at least in some fungal groups, because they have reached saturation and are possibly less informative than has been anticipated. Mycologists also encounter difficulties when analyzing DNA sequence data for many bitunicate fungi. In this case, even with *L. endophytica*, de Silva et al. [60] demonstrated that single gene phylogenies could reveal extensive incongruence (Figure 1, Figure 2 and Figure 3), which can be found in the supplementary information provided by de Silva et al. [60]. We could not compare the morphs of *L. fujianensisis* to *L. endophytica* as the latter was isolated as an endophyte and did not produce any fruiting bodies in culture. There is also a need to update the name of the GB accession numbers of MK501838, MK584572, and MK550606 as these are labelled as “*Lasiodiplodia sp. NIS-2019a isolate*”, but we presume that it should be *Lasiodiplodia endophytica*.

## Figures and Tables

**Table 1:** Isolates used in the phylogenetic analysis of *Lasiodiplodia* spp. and their GenBank accession numbers. Newly generated sequences are indicated in bold. * Type collections.

Species	Cultures	Host	Locality	Longitude and Latitude	GenBank			
					ITS	tef1-α	TUB	RPB2
*L. aquilariae*	CGMCC 3.18471	*Aquilaria crassna*	Laos	17°59′ N, 102°34′ E	KY783442	KY848600	N/A	KY848562
*L. avicenniae*	CMW41467	*Avicennia marina*	South Africa	25°44′ S, 28°15′ E *	KP860835	KP860680	KP860758	KU587878
*L. avicenniae*	LAS199	*Avicennia marina*	South Africa	25°44′ S, 28°15′ E *	KU587957	KU587947	KU587868	KU587880
*L. brasiliensis*	CMM 2321	*Carica papaya*	Brazil *	15°47′ S, 47°55′ W	KY783475	KY848612	KY848556	KY848595
*L. brasiliensis*	CMM 4015	*Mangifera indica*	Brazil *	15°47′S, 47°55′W	JX464063	JX464049	N/A	N/A
*L. brasiliensis*	CMW 35884	*Adansonia madagascariensis*	Madagascar *	18°52′ S, 47°29′ E	KU887094	KU886972	KU887466	KU696345
*L. bruguierae*	CMW41470	*Bruguiera gymnorrhiza*	South Africa *	25°44′ S, 28°15′ E	KP860833	KP860678	KP860756	KU587875
*L. bruguierae*	CMW42480	*Bruguiera gymnorrhiza*	South Africa *	25°44′ S, 28°15′ E	KP860832	KP860677	KP860755	KU587876
*L. caatinguensis*	CMM1325	*Citrus sinensis*	Brazil *	15°47′ S, 47°55′ W	KT154760	KT008006	KT154767	N/A
*L. caatinguensis*	IBL381	*Spondias purpurea*	Brazil *	15°47′ S, 47°55′ W	KT154757	KT154751	KT154764	N/A
*L. chinensis*	CGMCC3.18044	*Vaccinium uliginosum*	Shandong, China	36°03′ N, 120°22′ E	KX499875	KX499913	KX499988	KX499951
*L. chinensis*	CGMCC3.18061	unknown	Hainan, China	20°0′ N, 110°12′ E	KX499889	KX499927	KX500002	KX499965
*L. chinensis*	CGMCC3.18066	*Hevea brasiliensis*	Hainan, China	20°0′ N, 110°12′ E	KX499899	KX499937	KX500012	KX499974
*L. chinensis *	CGMCC3.18067	*Sterculia lychnophora*	Hainan, China	20°0′ N, 110°12′ E	KX499901	KX499939	KX500014	KX499976
*L. citricola*	IRAN1521C	*Citrus* sp.	Iran *	33°05′ N, 43°06′ E	GU945353	GU945339	KU887504	KU696350
*L. citricola*	IRAN1522C	*Citrus* sp.	Iran *	33°05′ N, 43°06′ E	GU945354	GU945340	KU887505	KU696351
*L. clavispora *	CGMCC 3.19594	*Vaccinium uliginosum*	Fujian, China	26°06′ N, 119°17′ E	**MK802166**	**N/A**	**MK816339**	**MK809507**
*L. clavispora *	CGMCC 3.19595	*Vaccinium uliginosum*	Fujian, China	26°06′ N, 119°17′ E	**MK802165**	**N/A**	**MK816338**	**MK809506**
*L. crassispora*	CMW 13488	*Eucalyptus urophylla*	Venezuela *	10°28′ N, 66°53′ W	DQ103552	DQ103559	KU887507	KU696352
*L. crassispora*	WAC12533	*Santalum album*	Australia *	32° S, 150° E	DQ103550	DQ103557	KU887506	KU696353
*L. curvata*	CGMCC 3.18456	*Aquilaria crassna*	Laos	17°59′ N, 102°34′ E	KY783437	KY848596	KY848529	KY848557
*L. curvata*	CGMCC 3.18476	*Aquilaria crassna*	Laos	17°59′ N, 102°34′ E	KY783443	KY848601	KY848532	KY848563
*L. euphorbicola*	CMM 3609	*Jatropha curcas*	Brazil *	15°47′ S, 47°55′ W	KU887149	KU887026	KU887455	KU696346
*L. euphorbicola*	CMW 33350	*Adansonia digitata*	Botswana *	24°36′ S, 25°40′ E	KU887187	KU887063	KU887494	KU696347
*L. euphorbicola*	CMW 36231	*Adansonia digitata*	Zimbabwe *	17°49′ S, 31°03′ E	KF234543	KF226689	KF254926	N/A
*L. exigua*	BL184	*Retama raetam*	Tunisia *	34°44′ N, 10°44′ E	KJ638318	KJ638337	N/A	N/A
*L. exigua*	CBS 137785	*Retama raetam*	Tunisia *	34°44′ N, 10°44′ E	KJ638317	KJ638336	KU887509	KU696355
*L. fujianensis *	CGMCC 3.19593	*Vaccinium uliginosum*	Fujian, China	26°06′ N, 119°17′ E	**MK802164**	**MK887178**	**MK816337**	**MK809505**
*L. gilanensis*	IRAN 1501C	Unknown	Iran *	33°05′ N, 43°06′ E	GU945352	GU945341	KU887510	KU696356
*L. gilanensis*	IRAN 1523C	Unknown	Iran *	33°05′ N, 43°06′ E	GU945351	GU945342	KU887511	KU696357
*L. gonubiensis*	CMW 14077	*Syzygium cordatum*	South Africa *	25°44′ S, 28°15′ E	AY639595	DQ103566	DQ458860	KU696359
*L. gonubiensis*	CMW 14078	*Syzygium cordatum*	South Africa *	25°44′ S, 28°15′ E	AY639594	DQ103567	EU673126	KU696358
*L. gravistriata*	CMM 4564	*Anacardium humile*	Brazil *	15°47′ S, 47°55′ W	KT250949	KT250950	N/A	N/A
*L. gravistriata*	CMM 4565	*Anacardium humile*	Brazil *	15°47′ S, 47°55′ W	KT250947	KT266812	N/A	N/A
*L. henanica*	CGMCC 3.19176	*Vaccinium uliginosum*	Shandong, China	36°03′ N, 120°22′ E	**MH729351**	**MH729357**	**MH729360**	**MH729354**
*L. hormozganensis*	IRAN 1498C	*Mangifera indica*	Iran *	33°05′ N, 43°06′ E	GU945356	GU945344	KU887514	KU696360
*L. hormozganensis*	IRAN 1500C	*Olea* sp.	Iran *	33°05′ N, 43°06′ E	GU945355	GU945343	KU887515	KU696361
*L. iraniensis*	CMM 3610	*Jatropha curcas*	Brazil *	15°47′ S, 47°55′ W	KF234544	KF226690	KF254927	N/A
*L. iraniensis*	CMW 36237	*Adansonia digitata*	Mozambique *	25°56′ S, 32°35′ E	KU887121	KU886998	KU887499	KU696348
*L. iraniensis*	CMW 36239	*Adansonia digitata*	Mozambique *	25°56′ S, 32°35′ E	KU887123	KU887000	KU887501	KU696349
*L. iraniensis*	IRAN 1502C	*Juglans* sp.	Iran *	33°05′ N, 43°06′ E	GU945347	GU945335	KU887517	KU696362
*L. iraniensis*	IRAN 1520C	*Salvadora persica*	Iran *	33°05′ N, 43°06′ E	GU945348	GU945336	KU887516	KU696363
*L. irregularis*	CGMCC 3.18468	*Aquilaria crassna*	Laos	17°59′ N, 102°34′ E	KY783472	KY848610	KY848553	KY848592
*L. laeliocattleyae*	BOT 29	*Mangifera indica*	Egypt *	30°03′ N, 31°14′ E	JN814401	JN814428	N/A	N/A
*L. laeliocattleyae*	CBS 130992	*Mangifera indica*	Egypt *	30°03′ N, 31°14′ E	JN814397	JN814424	KU887508	KU696354
*L. laosensis*	CGMCC 3.18464	*Aquilaria crassna*	Laos	17°59′N, 102°34′E	KY783471	KY848609	KY848552	KY848591
*L. laosensis*	CGMCC 3.18473	*Aquilaria crassna*	Laos	17°59′ N, 102°34′ E	KY783450	KY848603	KY848536	KY848570
*L. lignicola*	CBS 134112	dead wood	Thailand *	13°43′ N, 100°28′ E	JX646797	KU887003	JX646845	KU696364
*L. lignicola*	CGMCC 3.18460	*Aquilaria crassna*	Laos	17°59′ N, 102°34′ E	KY783462	N/A	N/A	KY848582
*L. lignicola*	CGMCC 3.18483	*Aquilaria crassna*	Laos	17°59′ N, 102°34′ E	KY783449	N/A	N/A	KY848569
*L. lignicola*	MFLUCC 11-0656	dead wood	Thailand *	13°43′ N, 100°28′ E	JX646798	N/A	JX646846	N/A
*L. macroconidica*	CGMCC 3.18479	*Aquilaria crassna*	Laos	17°59′ N, 102°34′ E	KY783438	KY848597	KY848530	KY848558
*L. macrospora*	CMM3833	*Jatropha curcas*	Brazil *	15°47′ S, 47°55′ W	KF234557	KF226718	KF254941	N/A
*L. mahajangana*	CMW 27801	*Terminalia catappa*	Madagascar *	18°52′ S, 47°29′ E	FJ900595	FJ900641	FJ900630	KU696365
*L. mahajangana*	CMW 27818	*Terminalia catappa*	Madagascar *	18°52′ S, 47°29′ E	FJ900596	FJ900642	FJ900631	KU696366
*L. margaritacea*	CBS 122065	*Adansonia gibbosa*	Western Australia *	31°56′ S, 115°55′ E	EU144051	EU144066	N/A	N/A
*L. margaritacea*	CBS 122519	*Adansonia gibbosa*	Western Australia *	31°56′ S, 115°55′ E	EU144050	EU144065	KU887520	KU696367
*L. mediterranea*	CBS 137783	*Quercus ilex*	Italy *	41°54′ N, 12°18′ E	KJ638312	KJ638331	KU887521	KU696368
*L. mediterranea*	CBS 137784	*Vitis vinifera*	Italy *	41°54′ N, 12°18′ E	KJ638311	KJ638330	KU887522	KU696369
*L. microcondia*	CGMCC 3.18485	*Aquilaria crassna*	Laos	17°59′ N, 102°34′ E	KY783441	KY848614	N/A	KY848561
*L. missouriana*	UCD 2193MO	*Vitis* sp.	USA *	38° N, 97° W	HQ288225	HQ288267	HQ288304	KU696370
*L. missouriana*	UCD 2199MO	*Vitis* sp.	USA *	38° N, 97° W	HQ288226	HQ288268	HQ288305	KU696371
*L. nanpingensis*	CGMCC 3.19596	*Vaccinium uliginosum*	Fujian, China	26°06′ N, 119°17′ E	**MK802167**	**N/A**	**MK816340**	**MK809508**
*L. nanpingensis*	CGMCC 3.19597	*Vaccinium uliginosum*	Fujian, China	26°06′ N, 119°17′ E	**MK802168**	**N/A**	**MK816341**	**MK809509**
*L. paraphysoides*	CGMCC 3.19174	*Vaccinium uliginosum*	Shandong, China	36°03′ N, 120°22′ E	**MH729349**	**MH729355**	**MH729358**	**MH729352**
*L. paraphysoides*	CGMCC 3.19175	*Vaccinium uliginosum*	Shandong, China	36°03′ N, 120°22′ E	**MH729350**	**MH729356**	**MH729359**	**MH729353**
*L. parva*	CBS 456.78	*Cassava field-soil*	Colombia, USA	34°0′ N, 81°1′ W	EF622083	EF622063	KU887523	KU696372
*L. parva*	CBS 494.78	*Cassava field-soil*	Colombia, USA	34°0′ N, 81°1′ W	EF622084	EF622064	EU673114	KU696373
*L. plurivora*	STE-U 4583	*Vitis vinifera*	South Africa *	25°44′ S, 28°15′ E	AY343482	EF445396	KU887525	KU696375
*L. plurivora*	STE-U 5803	*Prunus salicina*	South Africa *	25°44′ S, 28°15′ E	EF445362	EF445362	EF445362	EF445362
*L. pontae*	CMM1277	*Spondias purpurea*	Brazil *	15°47′ S, 47°55′ W	KT151794	KT151791	KT151797	N/A
*L. pseudotheobromae*	CBS 116459	*Gmelina arborea*	Costa Rica *	9°55′ N, 84°3′ W	EF622077	EF622057	EU673111	KU696376
*L. pseudotheobromae*	CGMCC 3.18047	*Pteridium aquilinum*	China *	39°54′ N, 116°23′ E	KX499876	KX499914	KX499989	KX499952
*L. pseudotheobromae*	CGMCC 3.18451	*Aquilaria crassna*	Laos	17°59′ N, 102°34′ E	KY783468	KY848621	N/A	KY848588
*L. pseudotheobromae*	CGMCC 3.18452	*Aquilaria crassna*	Laos	17°59′ N, 102°34′ E	KY783467	KY848620	KY848549	KY848587
*L. pseudotheobromae*	CGMCC 3.18453	*Aquilaria crassna*	Laos	17°59′ N, 102°34′ E	KY783460	KY848618	KY848545	KY848580
*L. pseudotheobromae*	CGMCC 3.18457	*Aquilaria crassna*	Laos	17°59′ N, 102°34′ E	KY783436	KY848613	N/A	N/A
*L. pseudotheobromae*	CGMCC 3.18461	*Aquilaria crassna*	Laos	17°59′ N, 102°34′ E	KY783446	N/A	N/A	KY848566
*L. pseudotheobromae*	CGMCC 3.18465	*Aquilaria crassna*	Laos	17°59′ N, 102°34′ E	KY783445	N/A	N/A	KY848565
*L. pseudotheobromae*	CGMCC 3.18466	*Aquilaria crassna*	Laos	17°59′ N, 102°34′ E	KY783444	KY848615	KY848533	KY848564
*L. pseudotheobromae*	CGMCC 3.18470	*Aquilaria crassna*	Laos	17°59′ N, 102°34′ E	KY783458	N/A	N/A	KY848578
*L. pseudotheobromae*	CGMCC 3.18474	*Aquilaria crassna*	Laos	17°59′ N, 102°34′ E	KY783452	N/A	KY848538	KY848572
*L. pseudotheobromae*	CGMCC 3.18475	*Aquilaria crassna*	Laos	17°59′ N, 102°34′ E	KY783459	KY848617	KY848544	KY848579
*L. pyriformis*	CBS 121770	*Acacia mellifera*	Namibia *	22°33′S, 17°04′E	EU101307	EU101352	KU887527	KU696378
*L. pyriformis*	CBS 121771	*Acacia mellifera*	Namibia *	22°33′ S, 17°04′ E	EU101308	EU101353	KU887528	KU887528
*L. rubropurpurea*	WAC 12535	*Eucalyptus grandis*	Australia *	32° S, 151° E	DQ103553	DQ103571	EU673136	KU696380
*L. rubropurpurea*	WAC 12536	*Eucalyptus grandis*	Australia *	32° S, 152° E	DQ103554	DQ103572	KU887530	KU696381
*L. sterculiae*	CBS342.78	*Sterculia oblonga*	Germany *	52°31′ N, 13°26 E	KX464140	KX464634	KX464908	KX463989
*L. subglobosa*	CMM3872	*Jatropha curcas*	Brazil *	15°47′S, 47°55′W	KF234558	KF226721	KF254942	N/A
*L. subglobosa*	CMM4046	*Jatropha curcas*	Brazil *	15°47′ S, 47°55′ W	KF234560	KF226723	KF254944	N/A
*L. tenuiconidia*	CGMCC 3.18449	*Aquilaria crassna*	Laos	17°59′ N, 102°34′ E	KY783466	KY848619	N/A	KY848586
*L. thailandica*	CBS 138653	*Phyllanthus acidus*	Thailand *	13°43′ N, 100°28′ E	KM006433	KM006464	N/A	N/A
*L. thailandica*	CBS 138760	*Mangifera indica*	Thailand *	13°43′ N, 100°28′ E	KJ193637	KJ193681	N/A	N/A
*L. theobromae*	CBS 111530	Fruit along coral reef coast	Papua New Guinea *	9°25′ S, 147°22′ E	EF622074	EF622054	KU887531	KU696382
*L. theobromae*	CBS 164.96	Unknown	Unknown	–	AY640255	AY640258	KU887532	KU696383
*L. tropica*	CGMCC 3.18477	*Aquilaria crassna*	Laos	17°59′ N, 102°34′ E	KY783454	KY848616	KY848540	KY848574
*L. venezuelensis*	WAC 12539	*Acacia mangium*	Venezuela *	10°28′ N, 66°53′ W	DQ103547	DQ103568	KU887533	KU696384
*L. venezuelensis*	WAC 12540	*Acacia mangium*	Venezuela *	10°28′ N, 66°53′ W	DQ103548	DQ103569	KU887534	KU887534
*L. viticola*	UCD 2553AR	*Vitis* sp.	USA *	38° N, 97° W	HQ288227	HQ288269	HQ288306	KU696385
*L. viticola*	UCD 2604MO	*Vitis* sp.	USA *	38° N, 97° W	HQ288228	HQ288270	HQ288307	KU696386
*L. vitis*	CBS 124060	*Vitis vinifera*	Italy *	41°54′ N, 12°18′ E	KX464148	KX464642	KX464917	KX463994
*Diplodia mutila*	CMW 7060	*Fraxinus excelsior*	Netherlands *	52°22′ N, 4°51′ E	AY236955	AY236904	AY236933	EU339574
*D. seriata*	CBS 112555	*Vitis vinifera*	Portugal	38°43′ N, 9°7′ W	AY259094	AY573220	DQ458856	N/A

**Table 2:** Tef1-α position of mismatch of *L. clavispora*, *L. gonubiensis*, *L. fujianensis*, *L. thailandica*, *L. paraphysoides* and *L. citricola*.

Species	Base Pair Difference	Nucleotides Difference (*tef1-α*)
*L. clavispora* and *L. gonubiensis*	A instead of G	30
	T instead of G	33
	T instead of gap	35, 36, 37
	G instead of gap	38, 42
	C instead of gap	39, 40, 41
	C instead of T	44, 48, 105
	G instead of A	121
*L. fujianensis* and *L. thailandica*	T instead of C	4
	A instead of G	7
	C instead of A	27
	gap instead of C	71, 74, 76
	gap instead of A	72
	gap instead of G	73, 75, 78
	gap instead of T	77
	C instead of T	92, 153, 296
	C instead of G	185
	G instead of C	495
*L. paraphysoides* and *L. citricola*	gap instead of A	9
	T instead of C	111
	gap instead of G	197
	A instead of G	248

## Data Availability

DNA sequence data generated in this study have been submitted to Genbank. Dataset used to generate phylogenies has been submitted to TREEBASE with accession number: 24322.
